# Synthesis of a novel highly active Ce(NDC)MOF@Bentonite nanocatalyst for sustainable production of hydrogen via the hydrolysis of sodium borohydride

**DOI:** 10.1038/s41598-026-59719-w

**Published:** 2026-06-29

**Authors:** Amira A. Mohamed, Fatma M. Dardir, Gehan T. El-Bassyouni, Abdalla M. El-Ayyat, Ezzat A. Ahmed

**Affiliations:** 1 Geology Department, Faculty of Science, Asiut University, Asiut, Egypt; 2 Refractories, Ceramics, and Building Materials Department, National Research Centre, Cairo, Egypt

**Keywords:** Bentonite, Ce(NDC)MOF, Nanocomposite, Sodium borohydride hydrolysis, Hydrogen Production, Environmental sciences, Chemistry, Materials science

## Abstract

One of the most important concerns now governing international attention is energy generation. In this work, Ce(NDC)MOF@Bentonite nanocomposite was synthesized and used as a novel and effective catalyst for the green synthesis of hydrogen. The synthesized composite was characterized using X-ray diffraction (XRD), thermogravimetric analysis (TGA), N_2_-adsorption, Fourier- transform infrared (FT-IR), and scanning electron microscope (SEM) techniques. On the view of the characterized data, a pure and distinct crystalline phase with a highly specific surface area (S_BET_) of 58.5 m^2^/g was formed. Moreover, the morphology of the nanocomposite was monitored through scanning (SEM). SEM image which revealed a highly porous surface texture with some large particles with highly spherical morphology. The catalytic performance of the fabricated catalysts was propped via the hydrolysis of sodium borohydride. The effect of NaBH_4_ concentration, weight of the catalyst, and the reaction temperature on NaBH_4_ hydrolysis was investigated. The hydrogen generation rate HGR of 116.02 mL min^−1^g^− 1^ at 30 °C was achieved using 16 mg of the catalyst and 0.05 M NaBH_4_. Kinetic and thermodynamic functions including activation energy, enthalpy, entropy, and free energy changed were also estimated. This work suggests that such novel and highly active catalysts hold strong potential as advanced materials for hydrogen energy applications.

## Introduction

One of the most pressing global challenges today is producing massive amounts of energy without significantly contributing to escalating environmental pollution^1^. The extensive reliance on fossil fuels has led to severe environmental degradation. Therefore, reducing fossil fuel dependency and promoting sustainable energy sources are considered both feasible and necessary strategies to mitigate this critical global issue^2^. Over the last two decades, scientists and engineers have devoted considerable research and development efforts toward renewable energy technologies^2^.

Hydrogen (H₂) is increasingly recognized as a vital element in the transition to sustainable energy systems and as a driver of green development^3,4^. It serves both as a feedstock and an energy carrier, making it a foundational component in future energy strategies^5,6^. According to the Hydrogen Council, H₂ could account for 18% of end-use energy demand by 2050, potentially reducing carbon emissions by 6 gigatonnes, generating US$2.5 trillion in revenue, and creating 30 million jobs worldwide^7^. However, realizing hydrogen’s role as a clean energy source requires attention to its method of production. Notably, stabilizing NaBH₄ in alkaline media slows down its self-hydrolysis, necessitating the use of efficient catalysts to enhance hydrogen generation from NaBH₄^8^.

Hydrogen is considered an ideal renewable energy carrier due to its high energy density, zero emissions, and abundant availability^9,10^. It can be produced through various methods broadly classified into two main categories. The first involves biomass-based technologies, including thermochemical^11^ and biological processes^12^. The second category comprises water-splitting techniques such as photoelectrolysis^13^, thermolysis^14^, and water electrolysis^15^. Nevertheless, hydrogen’s practical application is limited by storage challenges^16^. In contrast to pressurized hydrogen^17^ and cryogenic liquid hydrogen, materials with reversible hydrogen storage, such as metal/alloy hydrides^18^, carbonaceous materials^19^, and complex hydrides^20^, are most suitable^21^. Their high volumetric hydrogen capacity, acceptable energy efficiency, and safety features are mostly to blame for this. Furthermore, metal hydraid can be used to prepare hydrogen in a semi-continuous regime^22–25^. According to reports, this semi-continuous process provides steady generation rates without catalyst deactivation^26^.

Chemical hydrogen storage materials, such as NH_3_BH_3_^27–29^, NaBH_4_^30–32^, and magnesium composites^33–35^, have been the subject of increasing research interest due to their ability to store hydrogen effectively and safely. NaBH_4_ is one of the most researched of them because of its superior chemical stability, large hydrogen storage capacity, controlled hydrogen release, and high purity of the released hydrogen^30–32^. Despite this, the high cost can be a barrier to its current usage. Sodium borohydride (NaBH_4_) has many advantages including safety, relatively high stability, and containing 10 − 6 wt% of hydrogen^36,37^. The hydrolysis of 1 mol NaBH_4_ can provide 4 mol of hydrogen due to its large hydrogen storage capacity the hydrolysis of NaBH_4_ can proceed as manifested in the equation:

$$NaBH_{4} + {\text{ }}\left( {2{\text{ }} + {\text{ }}x} \right){\text{ }}H_{2} O{\text{ }} \to {\text{ }}NaBO_{2} \cdot xH_{2} O{\text{ }} + {\text{ }}4H_{2}$$

Metal-organic frameworks, or MOFs, are porous materials that combine organic and inorganic components^38–40^. Large surface area, high porosity, adjustable pore size, varied crystal structure (the Cambridge crystal database and CCD contain 60000–70000 distinct structures^41^, processing into thin films, and 3D products are just a few benefits that MOFs provide^42,43^. They can be used either as it is or after being modified post-synthesis. As a result, they have been employed as catalysts in a number of processes, such as the hydrolysis of NaBH_4_. MOFs and their derivatives were used as catalysts for the hydrolysis of NaBH4^44,45^. The materials can also be used as a precursor for the synthesis of an active catalyst via carbonization or pyrolysis, which preserves the morphology and porosity of the pristine MOFs. The metal centers of the frameworks can act as an active site for catalysis. The organic linker can be modified with metal to serve as a dynamic catalyst site. The pore can also be used to synthesize or stabilize functional nanoparticles. The carbonized materials can be used as it is or after the metal is removed through treatment, yielding a porous carbon skeleton with active sites for elements like phosphorus, oxygen, sulfur, nitrogen, and so on.

While mesoporous silica materials have been widely employed for hydrogen production, their high-cost limits large-scale use^46^ Therefore, exploring cost-effective, silica-rich natural materials like bentonite as alternative silica sources is a promising strategy.

This study builds upon previous investigations by focusing on synthesizing novel, active nanocomposite materials using low-cost, abundant bentonite as a silica support. The materials were crosslinked with organo-functionalized MOFs using naphthalene dicarboxylates as linkers. The catalytic efficiency of the resulting nanocomposites was evaluated through their performance in NaBH₄ hydrolysis for hydrogen generation.

## Experimental work

### Materials

A clay sample (Bentonite) was collected from Madamoud Formation, Gahdam area, Assuit, Egypt, as a natural source for metal organic framework composite. Cerium ammonium nitrate (Ce (NH_4_)_2_ (NO_3_)_6_), dimethyl formamide (DMF) (99.5% alfa chemicals), naphthalene dicarboxylates (NDC), and ethanol (99%) were used in the synthesis process. Sodium borohydride (NaBH4) was used as a source of hydrogen. All chemicals used are of high grade.

### Synthesis of Ce(NDC)MOF@Bentonite

To synthesize Ce(NDC)MOF@Bentonite the raw sample (bentonite) was crushed to powder. 1 g of bentonite was dissolved in 50 ml distilled water and stirred for 30 min. Subsequently, 2.9 g of ceric ammonium nitrate was dissolved in 8 mL distilled water and added to the former solution (mixture 1). Next 0.86 gm of naphthalene dicarboxylate (NDC) was dissolved in 32 ml dimethyl formamide (DMF) (mixture 2). In the next step, mixture 1 was added to mixture 2 and stirred for 24 h to form mixture 3. The last mixture was sonicated for 5 min. The mixture was transferred to the oven for one hour at 120 °C. After that, the product was washed with ethanol several times and separated using a centrifuge. Finally, dried at 70 °C for 24 h.

### Characterization technique

The mineral composition of both synthesized and raw materials was tested using the XRD technique. This depends on how the structure is measured. X-ray radiation with high energy and sensitivity. XRD patterns are produced by measuring the (2θ/degree) at which an X-ray beam with wavelength (λ) and intensity (I) is diffracted by the sample. The current test samples were XRD analyzed using the JSD-60/Joel diffractometers (Japan) at Assiut University using Ni-filtered Cukα radiation. The obtained diffractograms were indicated at 20 mA and 40 kV with a scanning rate of 5°/min in the 2Ɵ range of 4 to 60 2Ɵ.

The thermal behavior of the synthesized nanocomposite was studied utilizing a Linseis Thermal Analyzer (STA PT1600, Germany) in an airflow (40 mL/min). TGA/DSC analyses well done from abient room temperature till 700 ˚C with a heating rate of 5 ˚C/min.

A micrometrics instrument model ASAP 2010 (USA) was used to measure the adsorption and desorption of nitrogen at 196 ˚C. The samples were degassed for three hours at 110 C while under vacuum before analysis. The instrument software processed the adsorption data to compute the specific surface area using the BET equation, and the t-plot approach was used to determine the materials’ porosity using the Haisey equation. At P/P° = 0.95, the total pore volume, V, was computed. The BET and BJH methods were used to construct the pore volume distribution curve.

The chemical functional groups presented in the material under investigation were identified using a 6700 Nicolet Spectrophotometer (USA). The spectra of the substance were collected between 4000 and 400 cm^− 1^.The morphologies of the materials were examined using field emission scanning electron microscopy (FESEM) (FE-SEM; JEOL JSM7610F). With a JEOL (Germany) GmbH EDS X-ray photoelectron spectroscopic (XPS) analysis (model PHI 5000 Versaprobe) was conducted to study the chemical compositions of the prepared.

A transmission electron microscope (JEOL JEM-2100) running at an accelerating voltage of 200 kV was used to perform high-resolution transmission electron microscopy (HRTEM) and bright-field TEM pictures using mass-thickness contrast.

XPS spectra were recorded using (Thermo Scientific Escalab 250Xi, Thermo Fisher Scientific Inc., Waltham, MA, USA) with Al-Kα radiation (hν = 1486.6 eV).

### Catalytic activity

The sodium borohydride was hydrolyzed in a 100 mL round-bottom flask and the hydrogen gas that was produced as the only gas product was quantified using the conventional gasometric method. In order to equalize the pressure on the two sides of the manometer that are connected in series with the other parts of the system, the volume of hydrogen is measured and equaled to the volume of water displaced from the attached and normalized burettes. The flask was submerged in a magnetic stirrer equipped with an ultra-thermostat, which helped to maintain the desired temperature. A standard process involves injecting the right amount of catalyst into the flask along with 50 mL of NaBH_4_ (0.05 M) in deionized water to begin the catalyst’s activity. The formula R = v (mL) t^− 1^ m_cat_ (g), where v, t, and m stand for the volume of hydrogen generation, reaction time, and catalyst weight, respectively, was used to determine the rate of hydrogen evolution. These volumes were captured at various time intervals.

## Results and discussion

### Characterization

#### XRD analysis

**Fig. 1:**
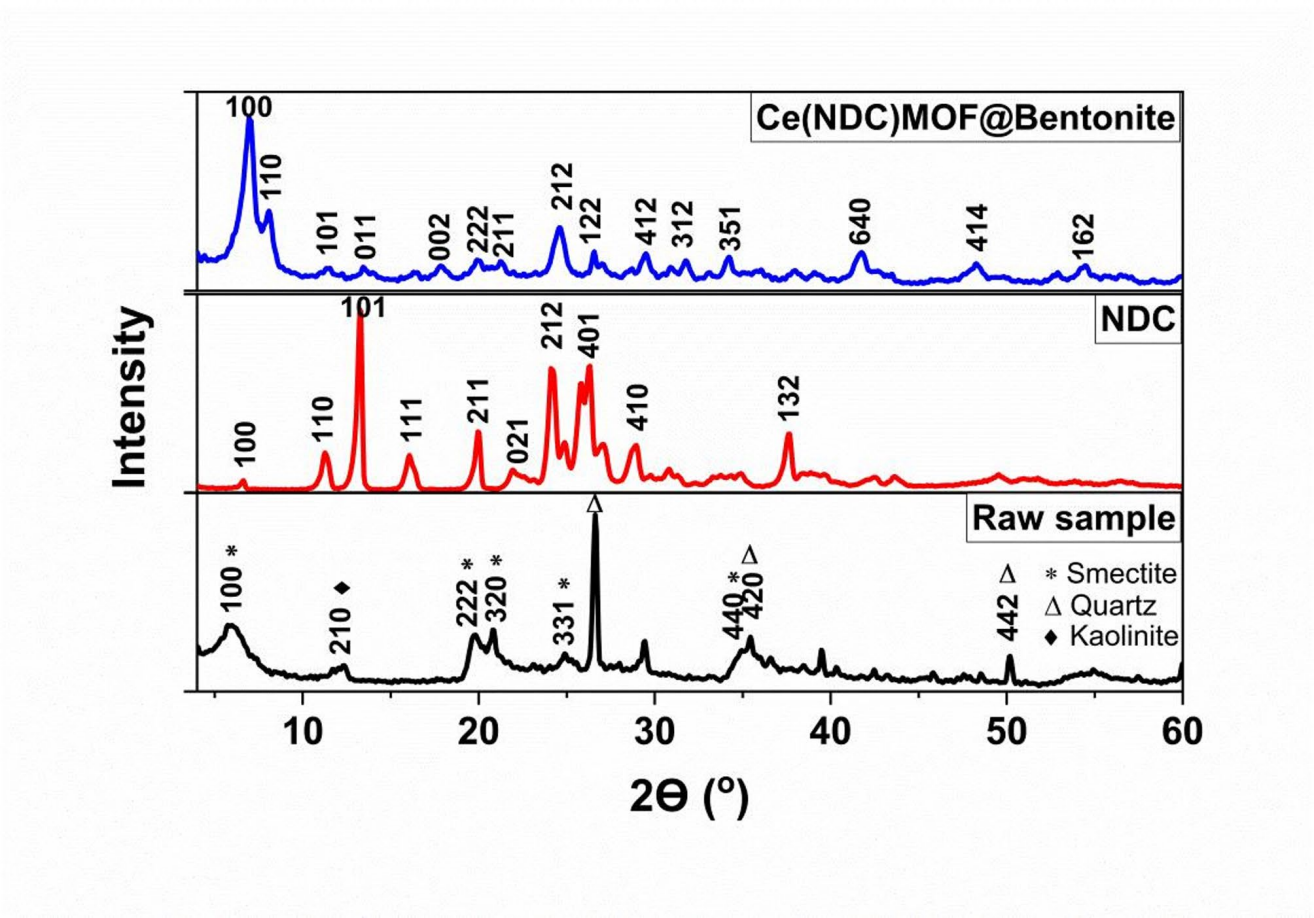
XRD of raw materials, NDC, and Ce(NDC)MOF@Bentonite.

X-ray diffractograms of raw sample, naphthalene dicarboxylate (NDC), and syntheized material were illustrated in Fig. 1. The x-ray diffraction pattern of the raw sample indicates the appearance of three main peaks located at 2Ɵ equal 5.8, 12.3, and 24.5 which are related to smectite, kaolinite, and quartz minerals respectively^47^. Meanwhile, the pattern of naphthalene dicarboxylate (NDC) reflects the crystalline well nature of the ligand used. The main peak corresponds to NDC appeared at 2Ɵ 13.24. on together with, the assigned of the peaks located at 2Ɵ 7, 8 revealed that such new composited is radially formed^48^. Further more, the peaks of the raw sample (bentonite) and naphthalene dicarboxylate (NDC) are disappeared. The synthesized MOF had an isostructural UiO-66 framework topology. Upon comparing and matching the different difractograms we can recongnize that, the Ce(NDC)MOF@Bentonite is completely formed.

#### Thermal analysis (TGA)

**Fig. 2:**
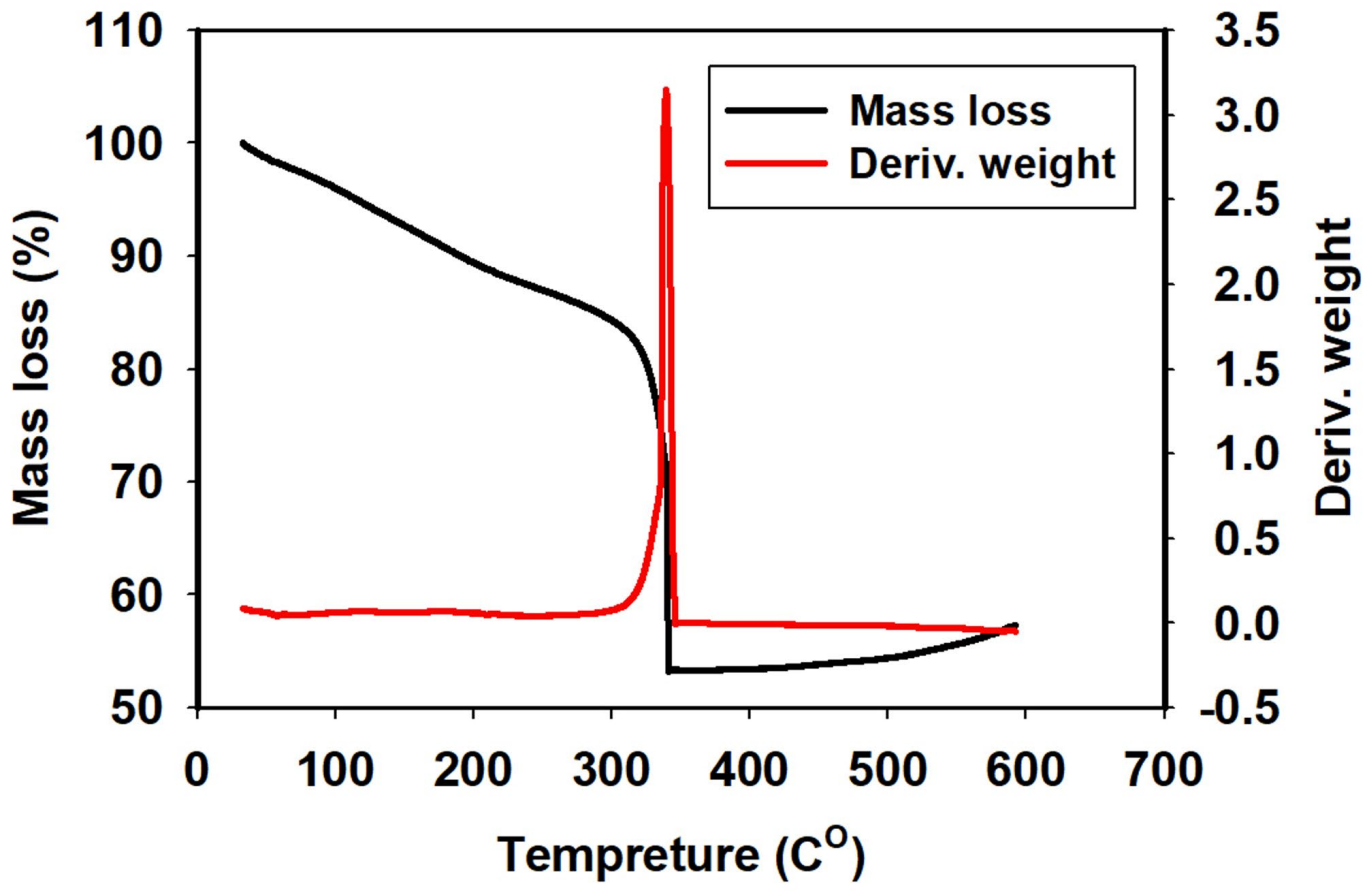
TGA curve of Ce(NDC)MOF@Bentonite.

To clarify the thermal changes accompanying the thermal treatment of the prepeared Ce(NDC)MOF composite, the TGA profile was carried out and given in Fig. 2. It demonstrates that there are three stages with a total loss of roughly 47% were seen when the MOF was heated from room temperature to 500 °C. The first stage of ~ 8% lies in the temperature range of 30–150 °C and is attributed to the evolution of adsorbed and hydrated water molecules^49^. The second stage is maximized at 198 °C and related to the evaluation of organic solvent used during synthesis process. On the other hand, the thired stage which startes at 240 °C, one can notice an abrupt weight loss amount to 40% at 315 °C that corespondes to the decomposition of the composite aleredy formed leading eventually to the corresponding oxide of cerium.

The differential thermogram (DTG) was constructed and inserted together with the TGA curve. From the DTG curve one can find a sharp peak located and maximized at 339 which matched with the noticeable abrupt drop in weight regarding to the main decomposition process of the MOF. Regarding the first stage belongs to evolution of both physical adsorbed water and the solvent existing in the temperature range was reflected on a broad ploten noticed on the DTG curve.

#### Texture studies

**Fig. 3:**
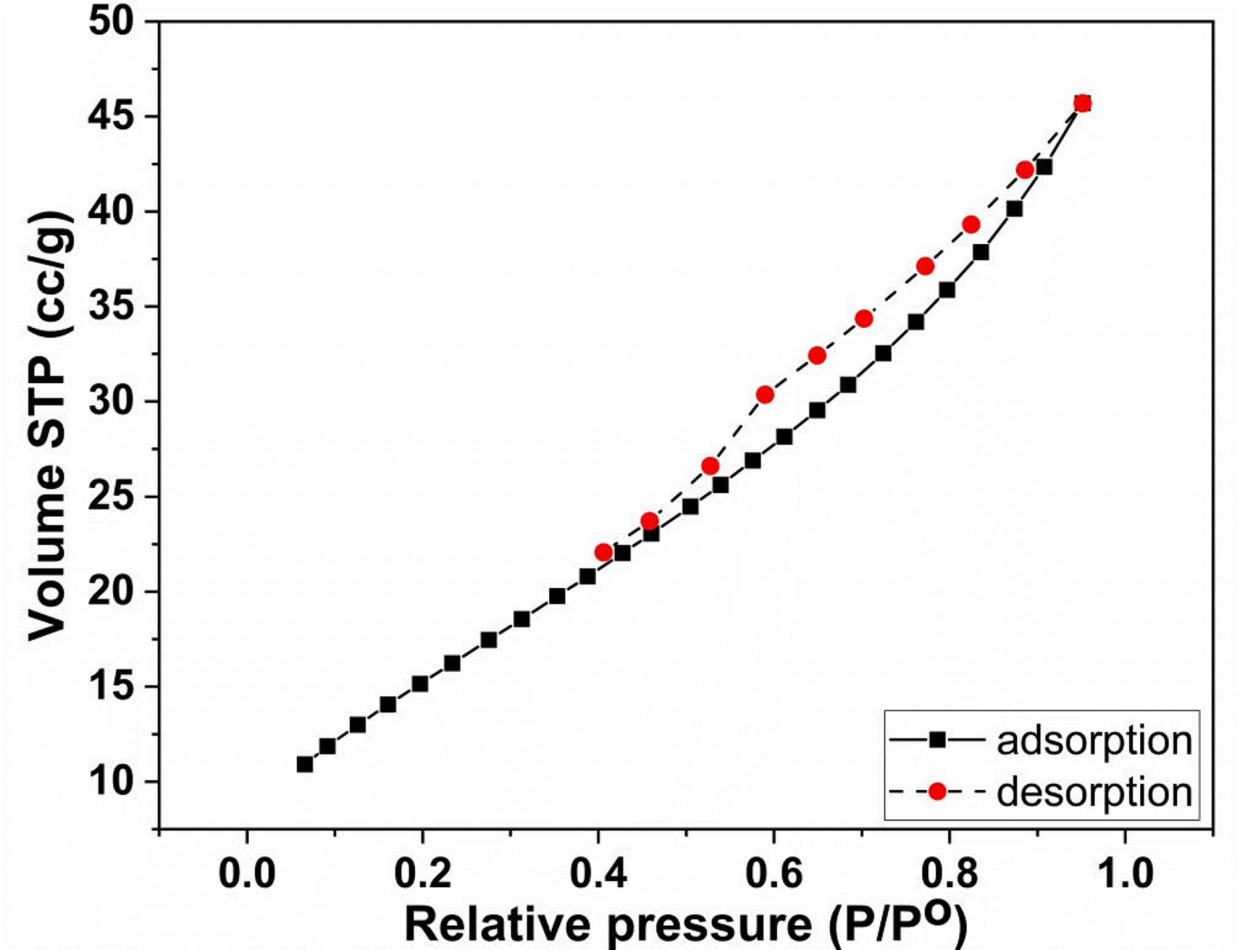
Adsorption-desorption isotherm of Ce(NDC)MOF@Bentonite.

**Fig. 4:**
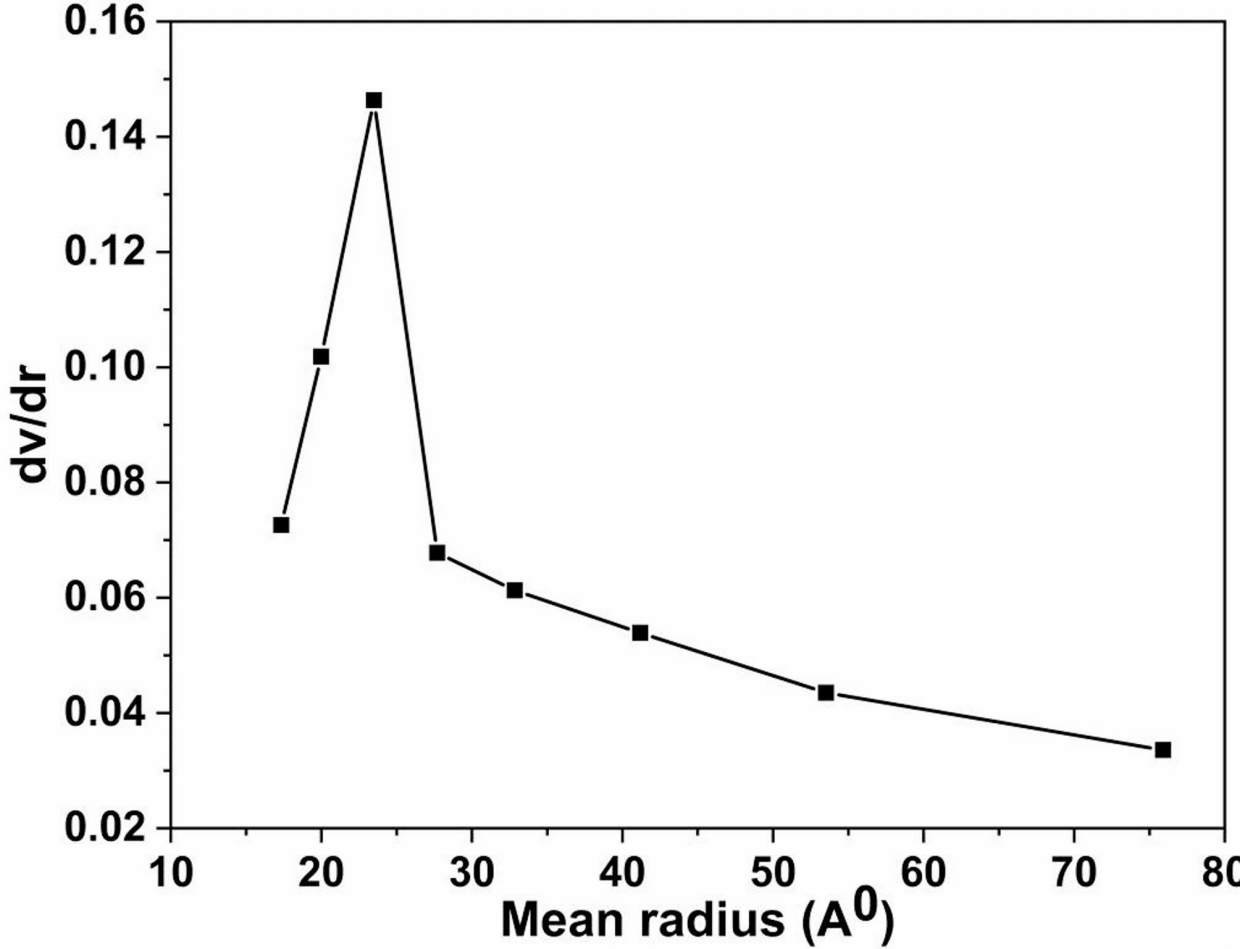
Pore volume distribution of Ce(NDC)MOF@Bentonite.

The N_2_- adsorption-desorption isotherm belongs to the nanocrystalline CeMOF composite was measured at 77 K and given in Fig. 3. The adsorption isotherm belongs to type IV with a little of type II of Brunaver’s classification^50^. This isotherm is distinguished by the existence of H_2_ and little of the H_3_ hysteresis loop. This behavior indicates that the MOF includes aggregates of the spheroical particles crossed by nearly cylindrical channels with non-uniform size and shape^51^ or agglomerates of particles forming slit shape pores. The specific surface area (S_BET_) of the material was calculated by using the BET equation in its typical applicability range of [P/P^0^ = 0.05–0.30] with a cross-sectional area of N_2_ = 16.2 Ao^52^–^54^. The examined surface area was found to be 58.5 m^2^/g. The porosity evaluation of the CeMOF composite was also investigated. The BJH method is used to create pore volume and surface area distribution^51,55^. The pore volume distribution curve is given in Fig. 4. The curve shows a peak located at r_p_ > 20A^0^ indicating the mesoporous nature of the nano-composite which are acceseable for adsorption of N_2_ molecules.

#### Fourier-transform infrared (FT-IR)

**Fig. 5:**
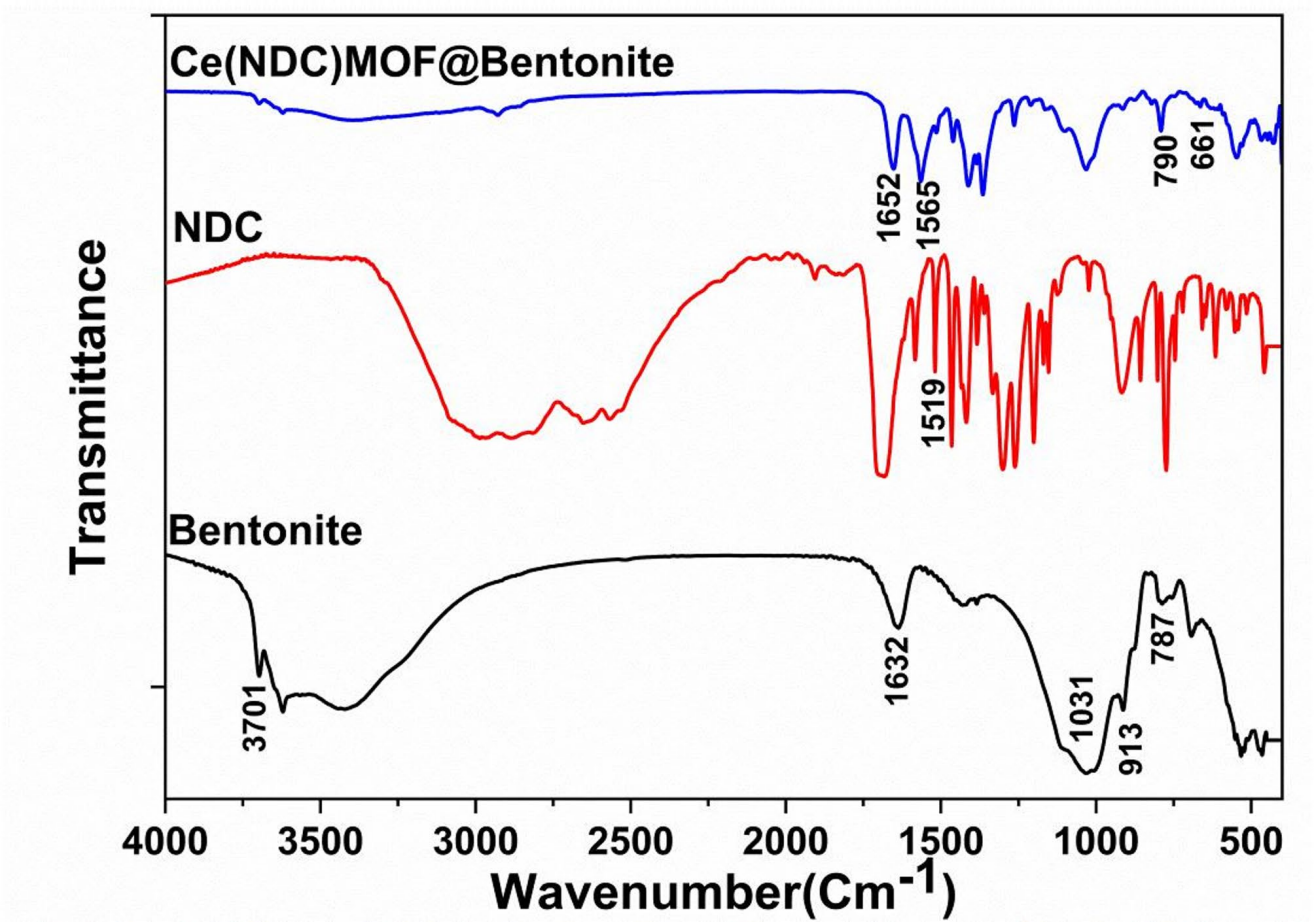
FT_IR spectra of raw bentonite, NDC, and Ce(NDC)MOF@Bentonite.

In spectroscopic techniques, the energy differences between the ground state and the excited one are analyzed. In the infrared region energy transitions due to the vibration of molecules groups are investigated. Therefore, the transform infrared (FT_IR) spectroscopy of NDC and its cross-linked material formed with cerium to have the corresponding Ce(NDC)MOF@Bentonite was applied to identify their functional groups. Moreover, FT-IR was measured for probing and confirming the chemical composition of the resulting composite. Inspection of the FT-IR spectra Fig. 5, the assignment of bentonite indicates a well-distinguished band at 3701 cm^− 1^ which can be attributed to the inner O-H groups located between the tetrahedral and octahedral sheets^56^. Additionally, it belongs to the stretching vibration of the inner surface to Al-OH. Meanwhile, the appearance of the bands at 3740 and 3650 cm^− 1^ suggested that the mineral is present in the organized state^57^. On the other hand, the symmetric stretching vibration of silicate tetrahedral is responsible for the band seen at 1031 cm^− 1^, while the band at 913 cm^− 1^ is linked to the Al-OH deformation vibration^58,59^. The Si-O symmetric vibration at 787 cm^− 1^ confirmed the presence of quartz. Figure 5, corresponds to NDC, the absorbance at 1519 cm^− 1^ is assigned for C = C stretching vibration. Where the other is related to the C-C band and C = O stretching vibrations. Furthermore, the FT-IR spectrum of Ce(NDC)MOF showed different disorganized bands characterized by the vibration of the Naphtalene ring which goes parallel with those given in the literature^60^. The doublet bands at 1652 cm^− 1^ and 1565 cm^− 1^ may be attributed to the in and out-phase stretching modes of COOH groups^61^. Finally, the bands assigned at 790 cm^− 1^ and 661 cm^− 1^ correspond to the OH and C-H present in the ligand part^62^.

#### Scanning electron microscope (SEM)

**Fig. 6:**
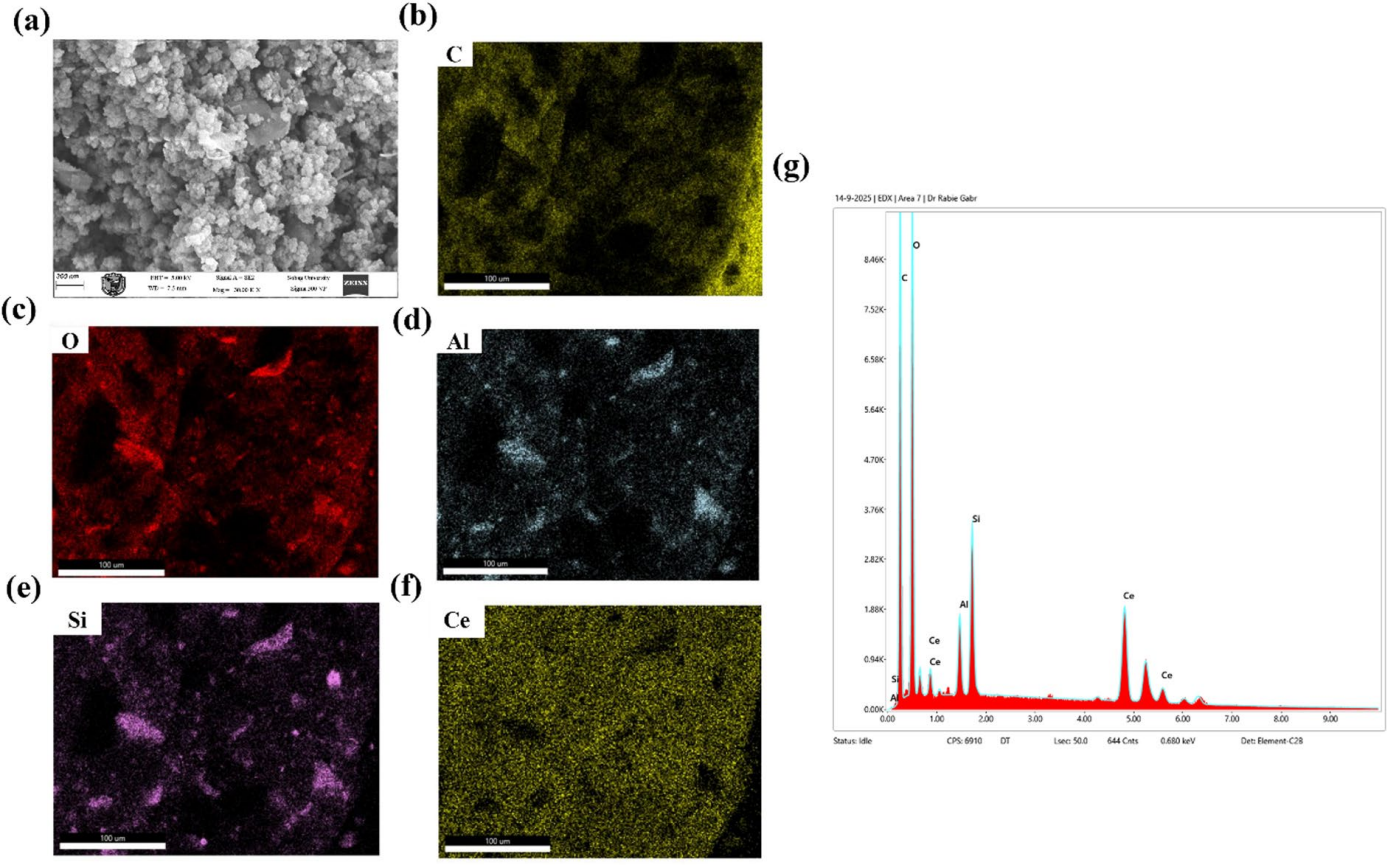
(**a**) SEM image of the synthesized nanocomposite. (**b**-**f**) EDX elemental mapping of C, O, Al, Si, and Ce. (**g**) The EDX spectrum.

The SEM image reveals the surface morphology of the Ce(NDC)MOF@Bentonite nanocatalyst, Fig. 6a displays a rough and porous structure with nanosized crystalline agglomerates distributed across the bentonite matrix. Such porous morphology is advantageous for catalysis, as it increases the surface area and enhances the accessibility of active sites. Figure (6 b-f) shows elemental mapping that confirms the elemental composition and spatial distribution of the nanocatalyst components: carbon (C, yellow) originates primarily from the organic linker (1,4-naphthalene dicarboxylate, NDC) in the Ce(NDC)MOF framework; oxygen (O, red) is present in both the bentonite aluminosilicate structure and the MOF carboxylate groups, supporting the successful integration of Ce(NDC)MOF with bentonite; aluminum (Al, gray) and silicon (Si, violet) are characteristic elements of the bentonite support, highlighting its aluminosilicate backbone; cerium (Ce, yellow) serves as the central metal ion of the MOF, and its broad, uniform distribution confirms the successful deposition and immobilization of MOF nanoparticles within the bentonite matrix. The mapping results demonstrate that all expected elements (Ce, C, O, Al, and Si) are well distributed and verifying the successful formation of the composite. The EDX spectrum further corroborates these findings by displaying distinct peaks corresponding to Ce, C, O, Al, and Si in Fig. 6g. The strong Ce signals validate the incorporation of Ce into the structure, while the Si and Al peaks confirm the contribution of the bentonite support, and the C and O peaks reflect contributions from both the bentonite and the MOF framework. Collectively, SEM, elemental mapping, and EDX analyses confirm the successful synthesis of the Ce(NDC)MOF@Bentonite nanocatalyst, characterized by its nanostructured porous morphology, uniform elemental distribution, and stable immobilization of Ce(NDC)MOF on the bentonite support—features that are crucial for enhancing catalytic performance.

#### Transmission electron microscope (TEM)

**Fig. 7:**
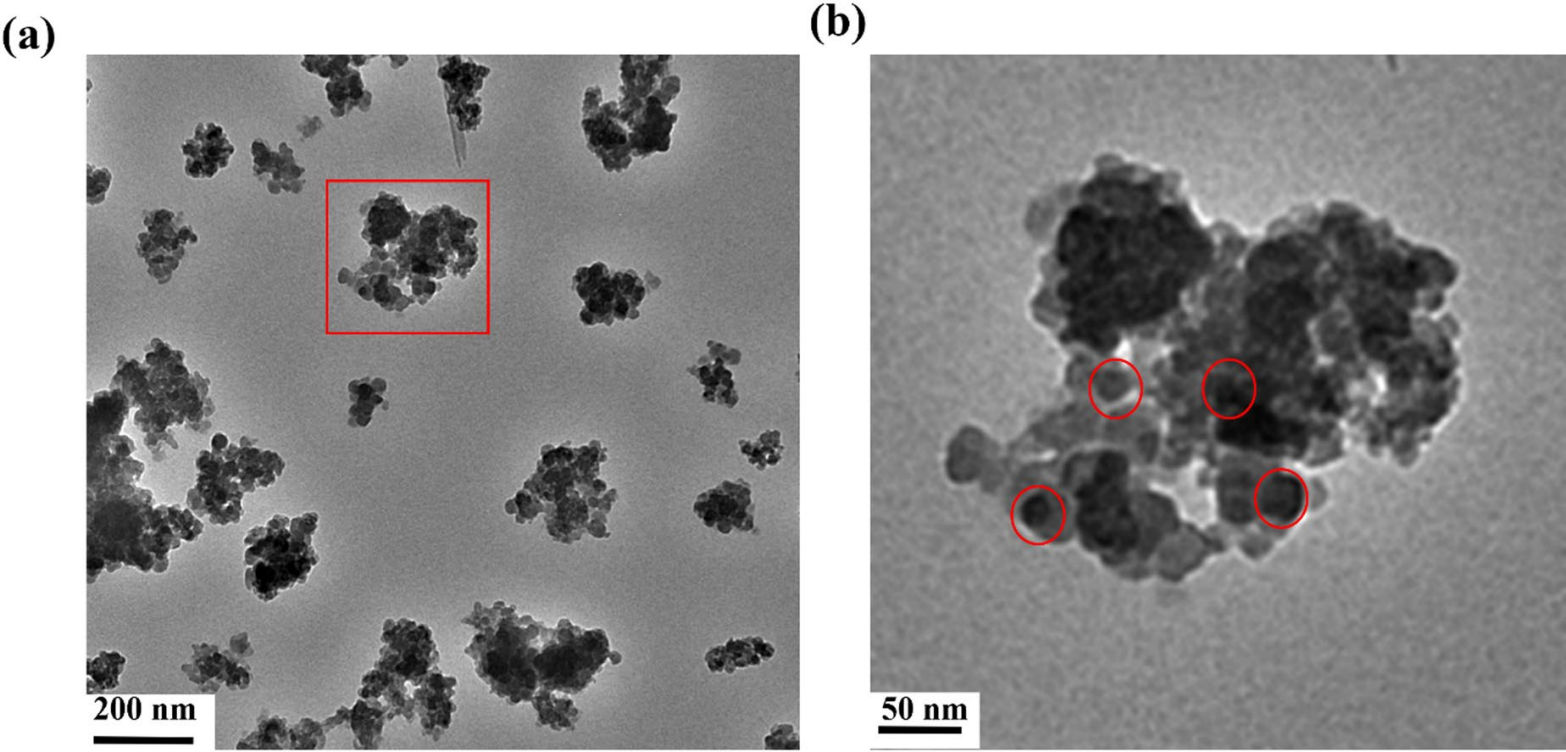
TEM images of the Ce(NDC)MOF@Bentonite nanocatalyst at different magnifications. (**a**) Low magnification. (**b**) High magnification.

Transmission electron microscopy (TEM) and high-resolution TEM (HRTEM) analyses provided detailed insight into the structural characteristics of the Ce(NDC)MOF@Bentonite nanocatalyst (Fig. 7a, b). The TEM image shows that the nanocatalyst exhibits randomly oriented, nearly spherical particles of varying sizes distributed on the bentonite support. The dark regions correspond to aggregated Ce(NDC)MOF crystallites, whereas the other lighter background represents the bentonite matrix. The particles appeared as irregularly shaped agglomerates, suggesting that the MOF crystallites are well-deposited and dispersed, although partial clustering is evident. The red box highlights a selected region magnified in Fig. 7b. At higher magnification, nanocrystalline Ce(NDC)MOF particles are clearly visible as dark, well-defined entities. The red circles mark individual Ce-containing particles with relatively uniform size distribution, confirming that the Ce(NDC)MOF crystallites are successfully immobilized on the bentonite surface. The crystallite size is estimated to be in the range of several tens of nanometers, verifying their nanoscale nature. Overall, TEM analysis confirms the successful incorporation and dispersion of Ce(NDC)MOF nanoparticles on the bentonite support, forming a composite nanocatalyst. The nanoscale crystallinity and homogeneous dispersion of MOF particles are expected to enhance catalytic performance by increasing the accessible surface area and the number of active sites.

#### X-ray photoelectron spctroscopy (XPS)

**Fig. 8:**
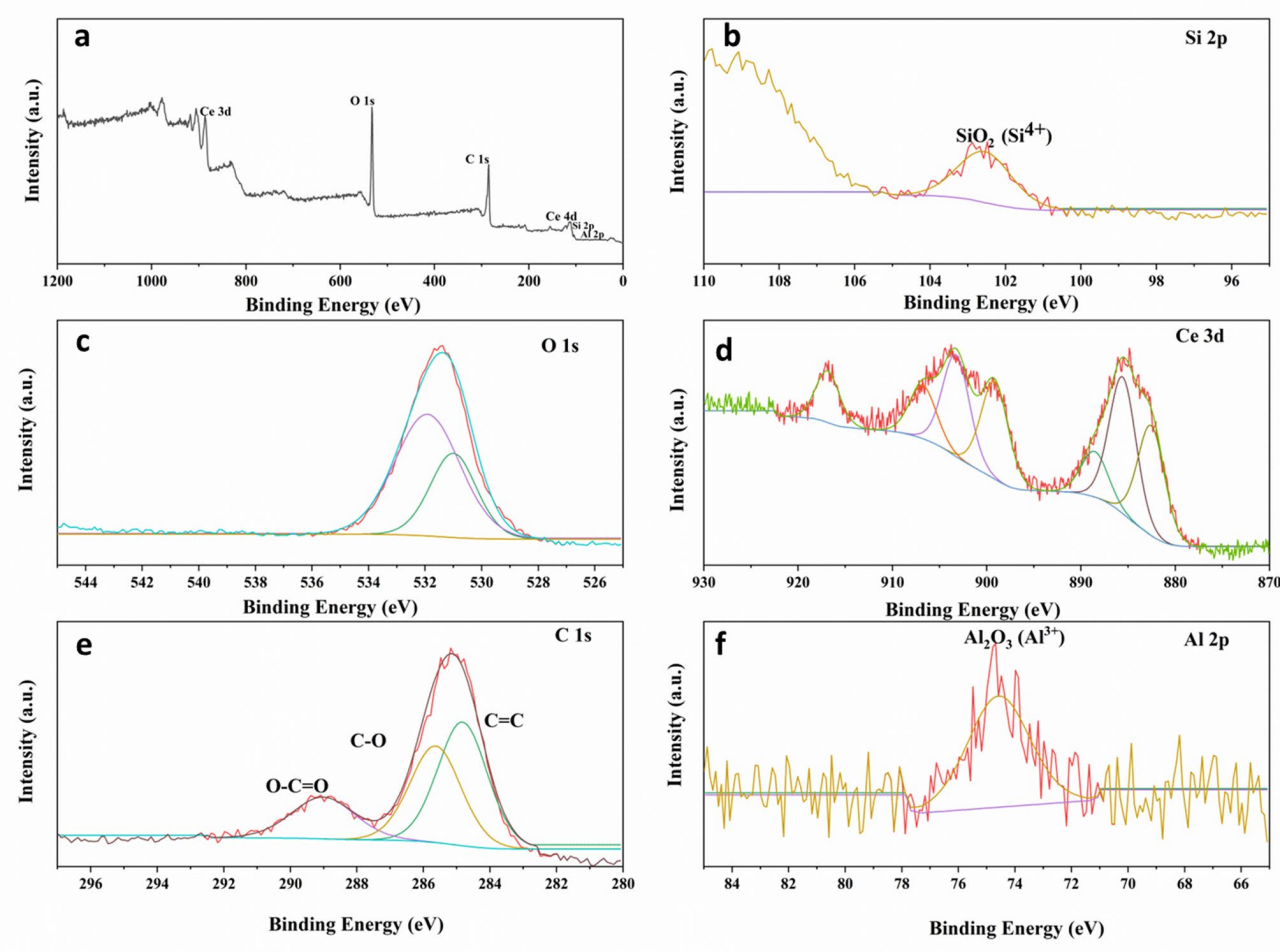
XPS spectrum analyses of Ce(NDC)MOF@Bentonite.

XPS spectra obtained for Ce(NDC)MOF@Bentonite nanocatalyst are presented in Fig. 8. The survey spectrum (Fig. 8a) monitors peaks indicative of Ce 3 d, O 1 s, C 1 s, Ce 4 d and a small Si 2p/Al 2p contribution from bentonite. The corresponding binding energies of the main peaks of the Ce 3 d spectra at 900 and 914.7 eV might be attributed to the Ce 3d5/2 and 3d3/2 peaks, respectively, for Ce(IV) (Fig. 8d), further, the peaks at around 878.4 and 903.2 eV observed in Ce 3 d spectra ascribed to Ce^3+^ 3d_5/2_ and Ce^3+^ 3d_3/2_, respectively^63^. Figure 8b shows the HR-XPS of Si 2p with one peak at 102.6 eV confirming the presence of Si^4+^ species in SiO_2_^64^. Conversely, the HR-XPS for Al 2p peak at 74.6 eV assignable to Al^3+^ in Al_2_O_3_ (Fig. 8f)^65^. The carbon C 1 s shows three peaks at 284.8, 285.6, and 289 eV referring to C-C, C-O, and O-C = O, respectively (Fig. 8e)^66^. O 1 s peak analysis resolves three peaks at 531, and 531.9 eV assignable are attributed to the lattice oxygen and surface adsorbed oxygen Fig. 8c, respectively^67^.

### Hydrolysis kinetics of sodium borohydride

The main goal when studying the kinetic behavior of the reaction is to have an idea about the mechanistic criteria of the given event. Though, we shall find that there is a wealth of many impacts affecting the reaction rate such as catalyst dose, the reactant concentration, and the reaction temperature. An understanding of these factors allows us to establish such criteria for predicting the reaction order and then go to the reaction mechanism. Although the catalytic efficiency is accelerated by increasing the reaction temperature, nevertheless the other reaction impacts can affect the reaction order. Consequently, the influence of the above-considered impacts should be studied.

### Effect of catalyst dose

**Fig. 9:**
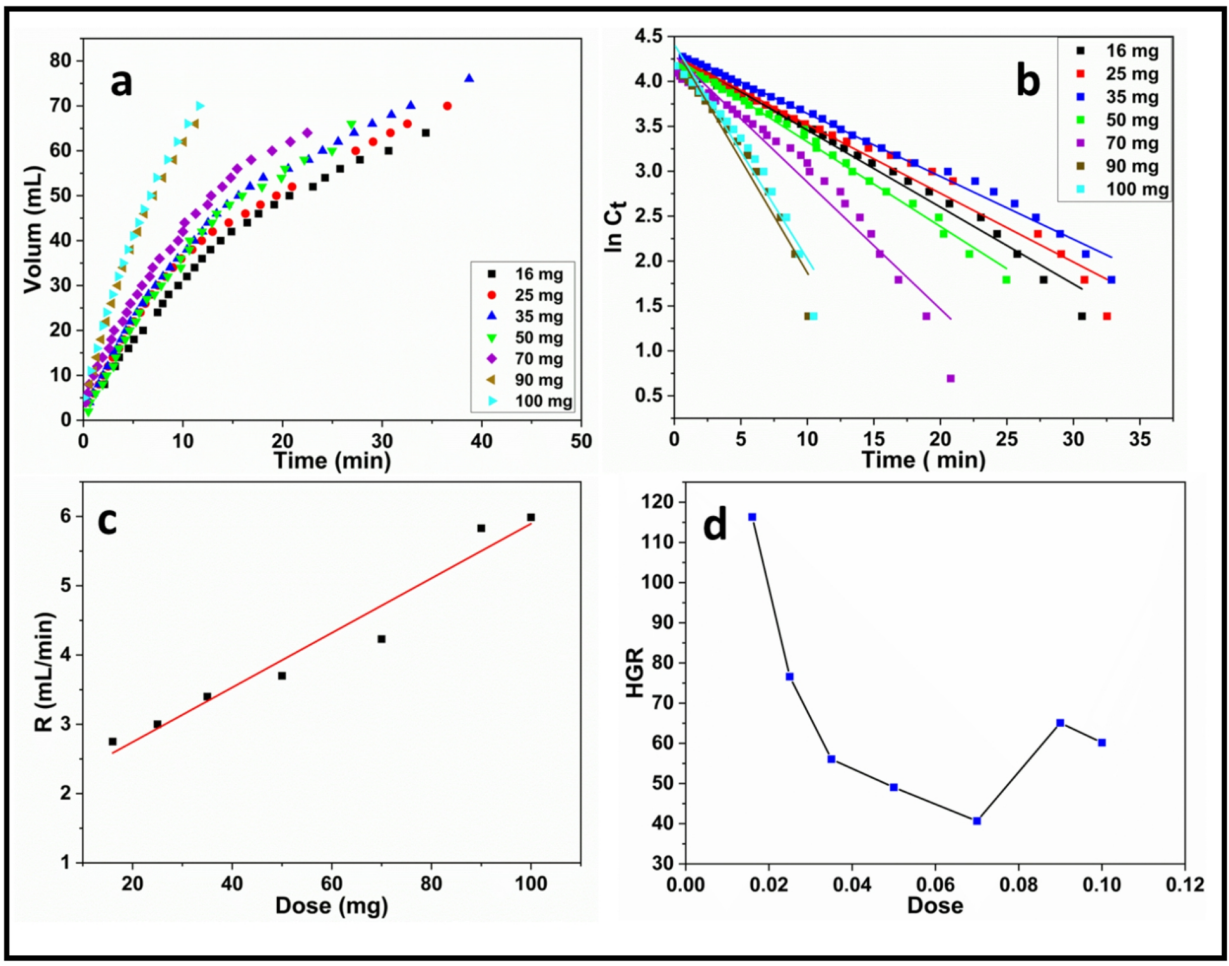
(**a**) Effect of catalyst dose on the hydrogen production, (**b**) first order plot at different catalyst doses, (**c**) variation of reaction rate with catalyst dose and (**d**) HGR vs. dose plot.

The influence of catalyst weight of CeMOF@Bentonite, on hydrogen production efficiency was investigated. In this study using 0.05 mol/L NaBH_4_ solution at 30 °C, in the presence of various catalyst weights ranging from 16 to 100 mg, the hydrogen volume was monitored throughout the entire reaction time. The obtained results are illustrated in Fig. 9a. It was believed that a catalyst weight controlled the process and was responsible for the hydrolysis of NaBH_4_^68^. The obtained curves in Fig. 9a revealed that when the catalyst weight is increased the reaction completion volume increases whereas the time of reaction completion decreases. These results prevailed that, the hydrogen evolution rate can be controlled by the catalyst dose. Additionally, using the trial and error method, the reaction order of n values was determined and found to be first-order kinetics when addressing the reaction order with regard to the borohydride^69–71^. The plotting between lnCt vs. time applied here verified the above results, where Ct stands for the hydride residual concentration at the whole time of measurement, the resulting graphs are displayed in Fig. 9b. The slope of the fitted lines of these plots provides the rate constant (K) value, while the intercept can be equalized to “ln a” where “a” is the initial volume of hydrogen produced. Results are displayed in Table 1. However, as shown in Fig. 9c, the computed hydrogen generation rate (R, mL/min) derived from the V-t plot can be plotted against the catalyst dose, the straight-line behavior shows a direct and linear proportionality between R and the catalyst dose. It also provides insight into the reaction order in relation to the catalyst dose.

The hydrogen generation rate (HGR) values were computed from the linear parts in the V_H₂_ - t plots and are given in Table 2. A continuous increase in HGR with reaction temperature which suggests that the reaction proceeds with the same mechanism and via the same active sites. On the other hand, the trend of variation of HGR with the catalyst mass indecate the reverse relation, thus a noticeable decrease in HGR with catalyst dose (Fig. 9d).

**Table 1:** Effect of catalyst dose on the reaction rate constant.

Catalyst dose (mg)	K (min^− 1^)
16	0.08563
25	0.07652
35	0.06998
50	0.09426
70	0.14122
90 100	0.25162 0.2389

**Table 2:** HGR with catalyst dose and tempertue.

**Catalyst dose(mg)**	**HGR**
16	116.33
25	76.60
35	56.06
50	49.04
70	40.66
90	65.08
100	60.17

**Temperature**(^o^C)	**HGR**
25	46.47
30	71.32
35	87.80
40	111.77
45	111.94

### Influence of sodium borohydride concentration

**Fig. 10:**
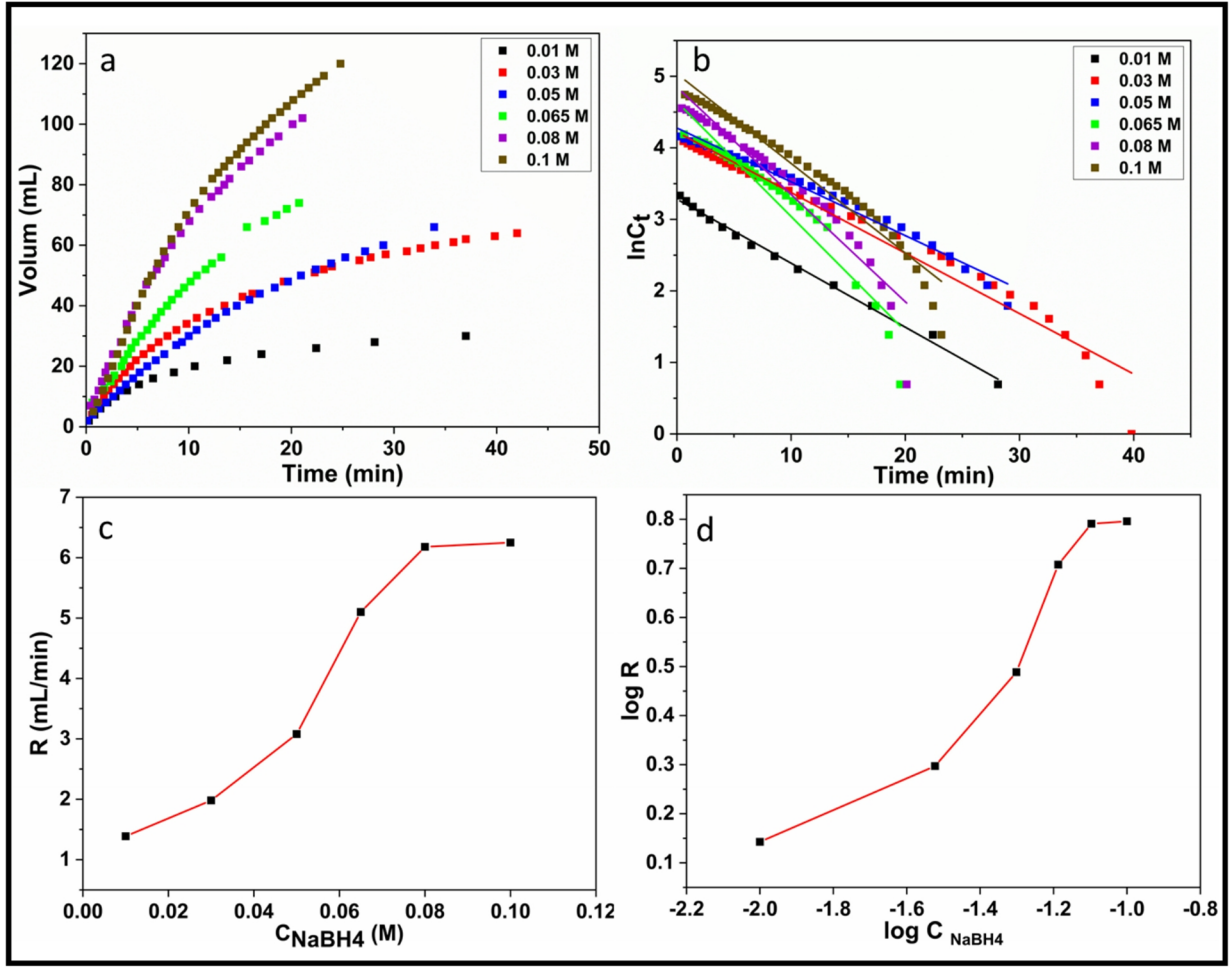
(**a**) Effect of NaBH_4_ concentration on hydrogen production, (**b**) variation of the remained concentration of NaBH_4_ measured with different NaBH_4_ initial concentrations, (**c**) variation of reaction rate with catalyst dose, and (**d**) logarithmic form of the relationship between reaction rate and NaBH_4_ concentration.

The effect of hydride concentration on the dehydrogenation reaction was investigated, such measurements were carried out at 30˚C using 50 mg of catalyst under various concentrations of NaBH_4_ (0.01–0.10 mol/L). Figure 10a shows the graphical presentation of the hydrogen volume as a function of time. It is clear that upon raising the concentration of NaBH_4_, the volume of hydrogen generated increases while the time required for full hydrolysis reduces. Additionally, Fig. 10b gives the linear logarithmic form of Ce vs. time, from each, we can calculate the corresponding hydrogen generation rate. There are a number of disparate results in the literature upon discussing the reaction sequence of NaBH_4_ at different concentrations. There are some publications^72,73^ conclude that first-order kinetics is the dominating one, while others^15,74^ stated that it is zero-order. The influence of different concentrations of NaBH_4_ on the hydrogen generation rate was examined in order to provide clear insight and deeper confirmation for such a study. The results are displayed in Fig. 10c. Inspection of the figure it is evident that the rises as the concentration of NaBH_4_ increases from 0.01 to 0.085 mol/L. However, when the concentration is increased further over 0.085 mol/L, there is no distinguished change in the rate. Furthermore, if we reconstruct our data on the way that log rate was plotted vs. log NaBH_4_ concentration Fig. 10d, thus we have two linear stages. The first one has a slope of 0.63, indicating first-order kinetics for the hydrolytic process (rate constant values are displayed in Table 3). The second stage possesses a slope of 0.05 when the hydride concentration is increased beyond 0.085 mol/L; as a result, the hydrolysis reaction exhibits zero-order kinetics. The above data highlighted the clear evidence that the reaction order is dependent on the hydride concentration and it change from 1 to 0 as the concentration of NaBH_4_ increases. To confirm the above mentioned results and gain a better understanding for the criteria of such occurrences, we have to consider few publications being conducted on the catalytic hydrolysis reaction of NaBH_4_^75,76^. These with our conclusions lead us to suggest that such a reaction proceeds through two involved processes: (1) preferential adsorption of BH^−^ _4_ anions on the surface of the catalyst active sites, and (2) hydrolytic reaction of the adsorbed species on the surface to produce hydrogen. Therefore, we would take the apportionety to suppose that at low NaBH_4_ concentration, the BH^−^ _4_ anions will partially occupy the more energetic centers at the catalyst support. While, at high concentrations, more of these anions existed at the bare active sites leading to more successive adsorption of such anions. However, when the hydride concentration is increased, BH_4_^−^ covers the entire catalyst surface, resulting in zero-order kinetics where the second process represents the rate-determining step.

**Table 3:** Effect of sodium borohydride concentration on the reaction rate constant.

Concentration of NaBH_4_ (mol/L)	K (min^− 1^)
0.010	0.0668
0.030	0.0716
0.050	0.0866
0.065	0.1050
0.080	0.1190
0.100	0.1250

### Effect of temperature

**Fig. 11:**
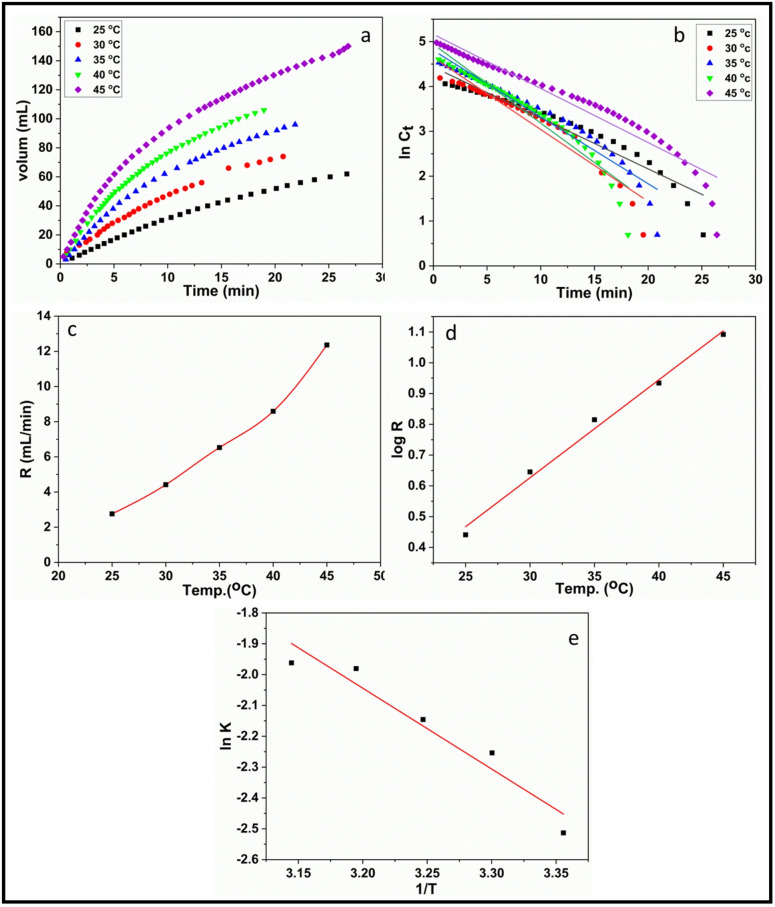
(**a**) Effect of temperature on hydrogen production, (**b**) corresponding first-order relation, (**c** and **d**) variation of reaction rate with temperature, and (**e**) representation of linear form of Van’t Hoff and Arrhenius equation.

In order to visualize the effect of temperature on the hydrolysis reaction, 50 ml of 0.05 mol/L NaBH4 solution together with 50 mg of the catalyst, then the reaction proceeded at a temperature ranging from 25 to 45 ˚C. The relationship between the volume of hydrogen and time at each temperature is represented in Fig. 11a. It is evident from the figure that the completion time of the hydrolytic reaction reterds with increasing temperature and increasing the rate of catalytic reaction (Fig. 11c&d). Furthermore, as the temperature rises, the volume of hydrogen increases, and the activity measurements were progressed. The plot of lnCt vs. t at various temperatures (Fig. 11b) justified the first-order rate constant values, which are listed in Table 4. Vant Hoff and Arrhenius Eqs. 3^77–80^ expresses the most significant relationship in chemical kinetics and also provides an abundant amount of information about the process that links the rate constant with temperature. The equation is given in Eq. (1).

1 $$K = {\text{ }}A{\text{ }}e\left( {{-}E/RT} \right)$$

Where K, R, A, T represent the reaction rate constant, universal gas constant (8.314 J mol^− 1^ K^− 1^), pre-experimental factor, and the reaction temperature, respectively.

Using the Arrhenius equation in its linear form (Eq. 2), the impact of temperature on the reaction rate was investigated in the temperature range of 25 to 45 ˚C. Plotting lnK values against the 1/T as given in Fig. 11e gives the straight line formation. Based on the slope of the resulting straight line, the activation energy was calculated and found to be 217.8 KJ/mol.

2 $$\ln {\text{ }}K{\text{ }} = {\text{ }}\ln {\text{ }}A{\text{ }} - {\text{ }}Ea/RT$$

The entropy of activation and free energy can also be calculated by using the subsequent Eyring’s Equation^81^.

3 $$K{\text{ }} = {\text{ }}kT/h{\text{ }}e^{{\Delta S/R}} ~e^{{\Delta H/RT}}$$

where K is the rate constant, k is Boltizman constant (10.381 × 10^− 23^ m^2^kg sec^− 1^), h is the Plank’s constant (86.626 × 10^− 34^ m^2^kg sec^− 1^), ∆s is the entropy change, ∆H is the enthalpy change, and T is the absolute temperature.

The relation between ∆H and Ea (∆E) can be given by the following equation:

4 $$\Delta H{\text{ }} = {\text{ }}\Delta E{\text{ }} + {\text{ }}\Delta nRt~$$

Where ∆ n is the difference between the number of moles of product and that reactants in their gaseous phases.

The thermodynamic relationship was terminated by the following equation:

5 $$\Delta G{\text{ }} = {\text{ }}\Delta H{\text{ }}{-}{\text{ }}T\Delta S~$$

Where ∆G is the change in Gibbs free energy.

Table 4 displays the corresponding ∆E, ∆H, ∆S, and ∆G values. The results given in Table 4 revealed that the estimated ∆G and ∆H possess negative values, whereas ∆S gives positive ones. These obtained thermodynamic functions and their signs indicate that the catalytic hydrolysis of NaBH4 on Ce(NDC)MOF@Bentonite is thermodynamically feasible and spontaneous.

**Table 4:** Kinetic and thermodynamic parameters were calculated for the hydrolysis reactions.

Temperature (kelvin)	K (sec^− 1^)	∆E (KJ)	∆H (KJ)	∆S (KJ)	∆G (KJ)
298	0.081	217.8	−220.32	1.00055	518.490
303	0.105	217.8	−220.36	0.98647	519.267
308	0.117	217.8	−220.40	0.97403	520.411
313	0.138	217.8	−220.45	0.9615	521.399
318	0.142	217.8	−220.49	0.95045	522.734

### Catalyst stability and reusability

**Fig. 12:**
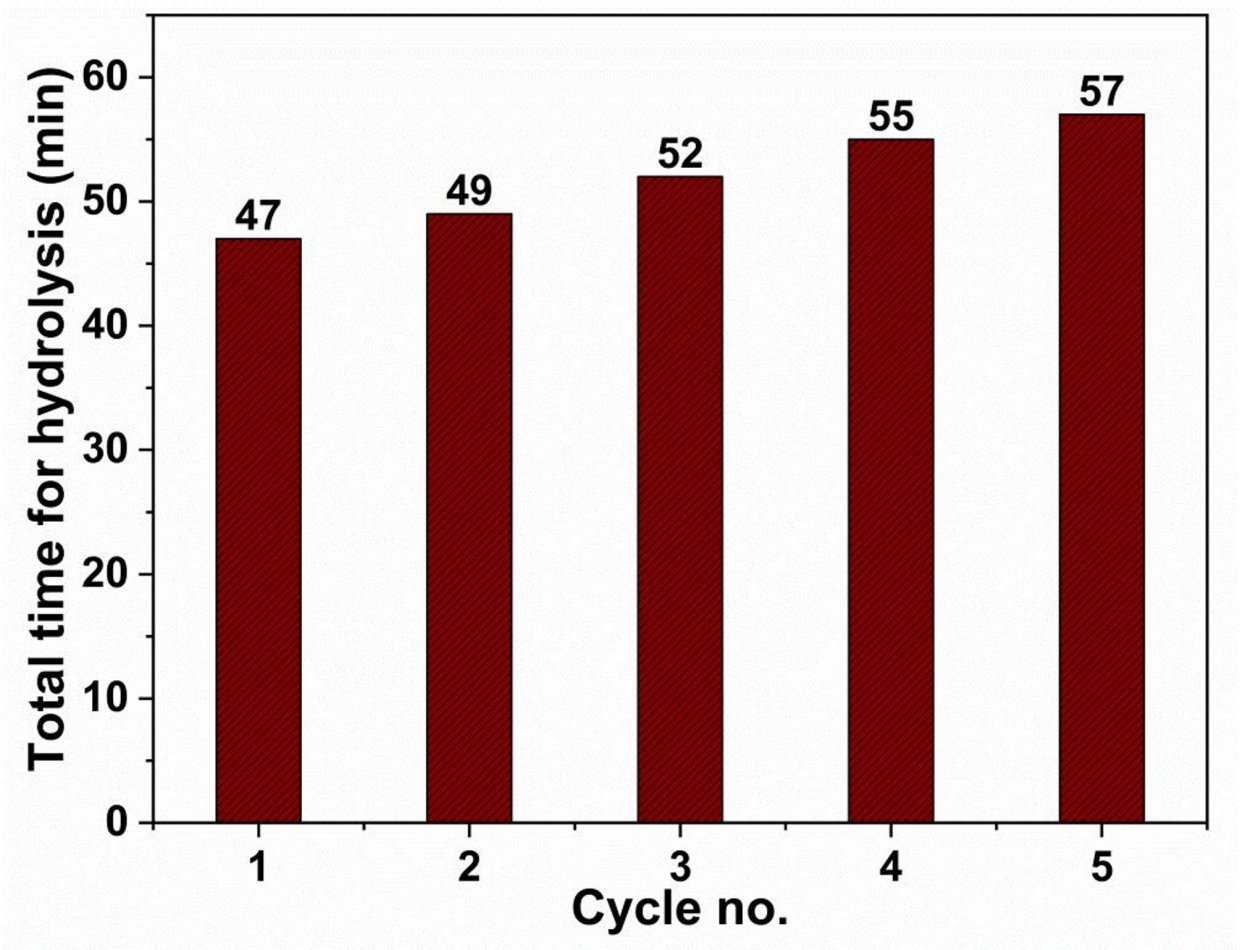
Reusability of 50 mg of CeMOF@Bentonite composite for hydrogen production from the hydrolysis of 0.05 mol/L NaBH_4_ at room temperature.

The practical use of catalysts in industrial hydrogen production depends critically on their reusability. In order to assess the catalyst’s reusability in the catalytic hydrolysis of NaBH_4_ (0.05 mol/L) with the Ce(NDC)MOF@Bentonite (50 mg), the catalyst was separated at room temperature following the hydrolysis, cleaned with distilled water, and then dried overnight to recover it for additional cycles. Figure 12 shows the results of our catalyst’s reusability test. The graphic makes it evident that when the catalyst is employed again, the amount of time needed to finish the hydrolysis reaction increases slightly. As a result, the rate of hydrogen creation noticeably declines. The decrease can be ascribed to either increased leaching of numerous active sites or the deposition of borate anions on the catalyst’s surface. As a result, during the next five runs, the catalyst activity steadily decreases with use.

Table 5 compares HGR values measured on our present catalysts with reported previously. Ce(NDC)MOF@Bentonite give higher HGR values than other catalysts when we used a littel amount of catalyst. They exhibited 116 mL.min^− 1^.g^− 1^ in 0.05 M NaBH_4_ at reaction temperature 30 °C. The comparison reveals that our catalysts are quite competitive and even superior to the reported catalysts in Table 3.

**Table 5:** HGR values measured using our present catalysts and literature-reported ones.

Catalyst	Catalyst weight	Reaction condition	HGR (mL.min^−1^.g^−1^)	Ref.
ZrOSO_4_/C-0 wt% H_2_SO_4_	100 mg	1 wt% NaBH_4_, room temperature	100	^82^
ZrOSO4/C-0 wt% H2SO4	100 mg	0.34 wt% NaBH_4_, room temperature	19	^82^
ZrOSO4/C-0 wt% H2SO4	100 mg	0.19 wt% NaBH_4_, room temperature	12.5	^82^
ZrOSO4/C-0 wt% H2SO4	100 mg	0.34 wt% NaBH_4_, room temperature	66.5	^82^
Ni	75%	14 wt% NaBH_4_, 30 °C	96.3	^83^
5.4 wt% Ru/Al2O3	500 mg	10 wt% NaBH_4_, 30 °C	65.5	^83^
Co/MWCNTs-2	15%	1 wt% NaBH_4_, 35 °C	109	^84^
Co-B/C	2.5%	1 wt% NaBH_4_, 5 wt%NaOH, 25 °C	166	^76^
Ce(NDC)MOF@Bentonite	16 mg	0.05 M NaBH_4_, 30 °C	116	This study

## Conclusion

In this article, the creation of a high noval and catalytic active nano-crystalline composite of Ce(NDC)MOF@Bentonite catalyst was performed. These catalysts were fully characterized through various physicochemical techniques. The catalytic performance of the synthesized materials were checked via the hydrolytic reaction of sodium borohydride. The effect of different impacts on hydrolysis rate and order of reaction were studied in order to have an idea about the criteria of both kinetic and mechanistic situations. The catalyst used monitored a noticeable hydrogen generation rate (HGR) of 116.02 ml min^-1^ g^-1^ and a low activation energy value of 217.8 KJ/mol which indicate that such nano-composite demonstrated excellent efficiency, rapid reaction time, minimal catalyst usage, and low mass leaching. The kinetic and thermodynamic calculations revealed the first-order kinetics at low concentrations of NaBH_4_. Moreover, the trend of variation in enthalpy, entropy, and free energy function shed light that the hydrolysis reaction is spontaneous, feasible, and exothermic in nature. Also, the synthesized composite exhibits noticeable recyclability, maintaining high catalytic performance over various consecutive cycles with minimal loss in efficiency. The observed data highlights the high activity of the catalyst and indicates its high potential for hydrogen-related application and suitability for large-scale industrial use.

## Data Availability

The data and chemical analysis present in the research article are available with the corresponding author (Fatma M. Dardir).
